# Covid-19 pandemic induced traumatizing medical job contents and mental health distortions of physicians working in private practices and in hospitals

**DOI:** 10.1038/s41598-023-32412-y

**Published:** 2023-03-31

**Authors:** Karl-Heinz Ladwig, Hamimatunnisa Johar, Inna Miller, Seryan Atasoy, Andreas Goette

**Affiliations:** 1 Department of Psychosomatic Medicine and Psychotherapy, Klinikum Rechts der Isar, Technische Universität München, Munich, Germany; 2 German Centre for Cardiovascular Research (DZHK), Partnersite Munich Heart Alliance, Munich, Germany; 3 Department of Psychosomatic Medicine and Psychotherapy, University of Gießen and Marburg, Gießen, Germany; 4 Global Public Health, Jeffrey Cheah School of Medicine and Health Sciences, Monash University Malaysia, Bandar Sunway, Subang Jaya, Selangor Malaysia; 5 Atrial Fibrillation NETwork (AFNET), Münster, Germany; 6 St. Vincenz-Krankenhaus GmbH, Medizinische Klinik II, Am Busdorf 2, 33098 Paderborn, Germany; 7 MAESTRIA Consortium AFNET e.V., Münster, Germany

**Keywords:** Psychology, Human behaviour

## Abstract

The Covid-19 pandemic during its early phases posed significant psychological threats particularly for medical frontline personal. It is unclear whether the medical workforce with the passage of time has adapted to these threats or have generalized to wider medical settings. An online survey was conducted reaching 1476 physicians in Germany with valid data from 1327 participants. Depression and anxiety were screened with the PHQ-2 and the GAD-2. Among a subtotal of 1139 (86.6%) physicians reporting personal treatment experiences with Covid-19 patients, 553 (84.8%) worked in a private practice (PP) and 586 (88.3%) in a hospital (HP). Covid-19 provoked profound conflicts between professional and ethical values: more physicians in PPs than HPs reported external constraints on their medical care being in conflict with the code of medical ethics (39.1 vs. 34.4%, *p* < 0.002) and significantly more HPs failed to maintain the dignity of their patients during the pandemic (48 vs. 27%, *p* < 0.0001). Comparison with reference groups among physicians with comparable size and settings during the first wave of Covid-19 revealed a significant increase in the prevalence of depression (23.0%) and anxiety (24.16%). Feelings of helplessness (63.3% in HPs and 53.4% in PPs) were associated with female sex, minor years of medical experience, sleeping problems and being encountered to unsettling events. Exposure to unsettling events and helplessness was significantly mediated by sleep disturbances (ß = 0.29, SE = 0.03, *p* < 0.0001). Covid-19 induced stress job content issues have broadened to medical disciplines beyond frontline workers. Emotional perturbations among physicians have attained a critical magnitude.

## Introduction

Shortly after the World Health Organization (WHO) qualified the newly identified SARS-CoV-2 virus spread as a “pandemic”, the majority of countries around the world initiated social distancing measures of a size never seen before. The rapidly evolving situation led to substantial economic setbacks and drastically disrupted the social and working life across the globe. Nevertheless, lockdown measures were only insufficiently able to slow down the spread of the SARS-CoV-2 virus subsequently leading to excess mortality and physical morbidity. Furthermore, it became rapidly clear that the Covid-19 pandemic also unfolded detrimental effects on mental health. Mainly based on large scale population based observational studies from countries around the globe conducted in the acceleration phase of the pandemic^1^, solid evidence accumulated elevated psychological distress, impaired wellbeing and Covid-19 induced increases in affective disorders (mainly depression, anxiety and posttraumatic stress disorders)^2^.

Among potential risk populations, particularly health care workers (HCW) with frontline personnel received attention in the public echoed by extended research activities. Early studies (e.g. a cross-sectional study performed 2019 in China including 1257 health care workers from 34 hospitals^3^) revealed that these professionals when exposed to Covid-19 have a high risk of developing unfavourable mental health outcomes and may need psychological support. An umbrella review, summarising the prevalence of anxiety and depression among HCWs during the Covid-19 pandemic, identified 10 systematic reviews based on evidence from 169,157 HCWs in 35 countries^4^. Mental health disruptions were pronounced: the prevalence of anxiety among physicians (n = 5820) ranged between 17% and 19.8% and for nurses (n = 14,938) between 22.8% and 27% while the prevalence of depression was significantly higher among physicians (40.4%) than among nurses (28%).

A closer look into pandemic induced disruptions of medical work organisations and its job contents was provided by a scoping review^5^ evidencing that the shift from patient-centred ethics in health care to public health-centred ethics—imposed by the current circumstances—triggered moral dilemmas and represented a major challenge in hospital settings. Of note, helplessness is a strong driver of mental health deteriorations as evidenced in a study with data from the general adult population^6^. However, the clinical importance of stress induced feelings of helplessness has not been investigated among medical professionals so far.

It seems that being in contact with affected patients, being young and thus being junior in terms of position at work, parenting children and having an infected family member are among risk factors of increased psychological distress commonly observed in HCWs during novel viral outbreaks^7^. Interestingly, a more recent study on the effects of work conditions and organisational strategies on nurses' mental health during the Covid-19 pandemic^8^ revealed that increased working hours, redeployment and occupational stigma were associated with adverse mental health and intention to leave for nurses, whereas caring for Covid-19 patients was associated with a lower risk of adverse mental health after adjustment for other work conditions.

The wear and tear of the pandemic resulted in several waves. In the second half of the year 2021, Europe faced a fourth infection wave, thus accumulating adverse traumatic burden of face-to-face experiences and sustained threat for subjects working in medical settings. On the other hand, psychological adaptation may have taken place—a phenomenon often observed among emergency medicine personal^9^. Furthermore, the focus on frontline workers may have obstructed the view on other medical settings, particularly on personnel in ambulances. Despite clear statements on the impact of the Covid-19 pandemic on physicians working in private practises, research on this topic compared to hospital staff is still in its infancy. D. Kamerow, for example, forcefully remarked 2022 in a commentary for a scientific journal that *“…dramatic changes in practice have come very quickly, without much prior preparation. Little assistance, or even clear guidance, has come from authorities*”^10^. Lasalvia et al.^11^ analysed the psychological impact of Covid-19 among 215 General practitioners (GPs) in Italy during the first pandemic wave and found that 44.7% reported Covid-19-related traumatic events. Among these, 36% (95% CI, 26‒46%) developed symptoms of post-traumatic distress. Being female, working in rural settings, and having less professional experience were associated with higher anxiety and depression. The ability to diagnose Covid-19 increased self-perceived professional efficacy, thus contributing to burnout reduction. In discussing their findings, Lasalvia et al.^11^ stated that GPs risked significant exposure to the SARS-CoV-2 infection, visiting a large number of patients often directly in their homes, with minimal control over their work environment. Extensive involvement in end-of-life care, traumatic events (such as death and dying) combined with the task of making onerous decisions, feelings of futility, and being forced to practise outside their areas of clinical expertise, may have exposed GPs to an increased risk of adverse psychological outcomes. It does not come to a surprise that a French national online cross-sectional survey including 1992 GPs disclosed psychological distress in 76.8% outpatient physicians in private practice. Covid-19 induced distress was associated with higher psychotropic drug use in the last twelve months, or increased alcohol or tobacco consumption due to work-related stress^12^. Recently, Collins et al.^13^ analysed mental health among 3711 GPs/family physicians in 33 countries during Covid-19 and revealed that almost 65% of respondents were at risk of distress. GPs with less experience, in smaller practices, and with more vulnerable patient populations were at a higher risk. To the best of our knowledge, the only study addressing the different impact of hospital settings compared to private practice settings has been performed in a French investigation among radiologists^14^. Against expectation, they disclosed that working in a public hospital was a *protective* factor for insomnia, anxiety, and depression (OR 0.4 [95%CI 0.2–0.7], 0.6 [0.4–0.9], and 0.5 [0.3–0.8]).

Therefore, the purpose of the present investigation was 1) to assess systematically the traumatogenic job content in the professional life of physicians during the fourth wave of the Covid-19 pandemic course and 2) to elucidate the differential impact of the pandemic on the mental distress of physicians working in hospitals (HPs) and in private practices (PPs). Beyond symptoms of anxiety and depression, limited information is available on the impact of personal, job-related, organizational, and Covid-19-related factors including possible ethical dilemmas on mental health outcomes among hospital physicians and GPs. Assessment of these conditions is a second major study aim of the present investigation.

## Methods and participants

### Setting

The present study was conducted as online survey distributed by the local Medical Council of a western district of Germany (Westfalen-Lippe) and operationally supported by the academic research organisation Atrial Fibrillation NETwork (AFNET) in Münster (NRW) covering a catchment area of 8.2 Mio inhabitants. The study was approved by the Ethics Committee of this Medical Council together with the University of Muenster on September 16th 2021. All methods were carried out in accordance with relevant guidelines and regulations. Informed consent was obtained from all subjects and/or their legal guardian(s). The campaign was launched in a period of two months between November 4th and December 31st 2021. The survey was anonymous, and confidentiality of information was ensured. No reply emails were provided.

### Participants

**Figure 1:**
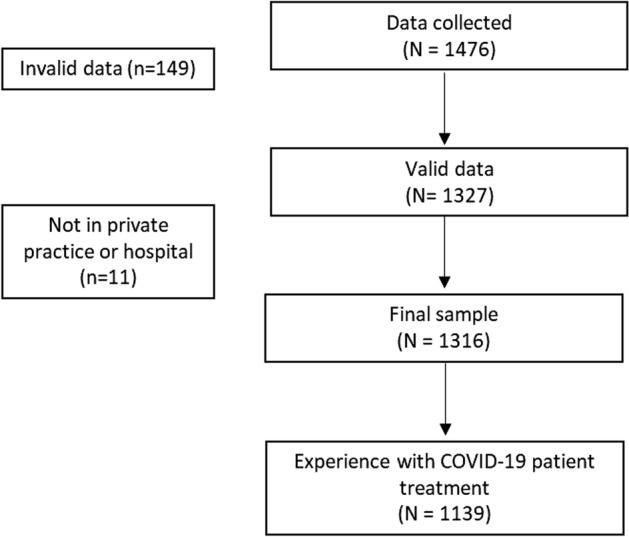
Study flowchart.

Participants from the following disciplines were invited: general, internal and paediatric medicine, general surgery, gynaecology. Originally based on 1316 valid cases, a total of 1.139 participants reported treatment experiences with Covid-19 patients, thus qualifying as the index group for this study (Fig. 1).

### Survey measures

#### Basic data

The survey documented the participant’s sex; working in PP or in HP; years of professional experience (coded threefold) and whether the participant had experience in treatment of Covid-19 patients. The participants’ vaccination status was documented and whether he/she had been already affected by Covid-19.

#### Pandemic induced traumatogenic impact of the medical job content

We assessed the job content items through a modified Delphi process (under the lead management of A.G.) utilising the experience of professionals working in the field to identify quality indicators for evaluating Covid-19 induced job stress items first by gathering items, and in a second round shortening the list through prioritisation leading to nine items reflecting adverse working conditions during the pandemic covering the following content: confrontation with dying patients (0–3); number of Covid-19 patients treated (0–3); professional restrictions (0–1); forced to limit treatment of non-Covid-19 patients (0–1); external constraints on medical care in conflict with the code of med. ethics (0–3); inability to maintain the dignity of patients (0–3); forced to prioritize treatment options (0–3). In order to capture the pandemic induced traumatizing impact of the medical job content, we combined these nine items to a general score ranging from 0 to 17. For the score variable, there were 176 missing data.

#### Future outlook of the pandemic

To seize the impact of the Covid-19 Pandemic on future medical treatment, participants were asked whether the pandemic will have a lasting effect on medical care, whether telemedicine will receive an increasing meaning in medical treatment and whether he/she will offer telemedicine-applications him/herself? The psychological aspect of future outlook was assessed by rating the personal future on a VAS scale 1–10, ranging from catastrophic to excellent.

#### Behavioural and psychological variables

Feeling helpless: Helplessness is defined as a profound belief that control over the situation or its outcomes is impossible. Following a suggestion by Shea and Barney^15^, this factor was measured by a dichotomized one-item question (“*How helpless did you feel during the pandemic*?”). Self-rated health (SRH) was assessed by a one standardized question recommended by the WHO asking participants to rate their current health on a 5-point scale in response to the question “Do you consider your health at the moment to be very poor, fair (average), good, or excellent?” For the analyses, we used a 3-point scale (good, fair, poor SRH) by aggregating excellent and good SRH, and very poor and poor SRH^16^. Sleep disturbances were assessed based on three items adopted from the Uppsala Sleep Inventory^17^ concerning difficulties initiating sleep (DIS), difficulties maintaining sleep (DMS) and premature awakening each scoring 0 to 1, leading to score range of 0–3. Depression and general anxiety symptoms were measured with the short form of the Patient Health Questionnaire (PHQ–D) which is divided into a depression (PHQ-2) and a generalized anxiety module (GAD-2) (both with two items ranging from 1 to 3). We considered a cut-off-value of ≥ 3 as indicative for probable cases of clinically significant levels of depressive and anxiety symptoms. Psycho-traumatogenic impact was assessed employing a one-item question with a Likert-like scoring ranging from 0 to 5 *(“…to which degree have encounters with Covid-19 patients afflicted you?”).*

#### Statistical analysis

Participant’s characteristics were stratified by PP and HP settings and presented as proportions or means ± standard deviation (± SD), accordingly. Bivariate associations between groups were tested using the χ2 test for categorical variables and Wilcoxon–Mann–Whitney test for continuous variables. Comparisons with reference values were performed with the one sample t-test for continuous variables. Means (± SD) and *p* values for pairwise comparison were reported as were effect sizes (Cohen’s d) (d ≥ 0.2 = small, d ≥ 0.5 = medium, d ≥ 0.8 = large effect size)^18^. Multivariate linear regression analyses were performed to examine factors associated with helplessness among participants (N = 1315).

We evaluated the mediation effect of sleep disturbances on the association between unsettling events and helplessness within a four-step framework described by Baron and Kenny^19^ Step 1 examined the relationship between unsettling events and sleep problems (path a). Step 2 examined the relationship between sleep problems and helplessness while controlling for unsettling events (path b). Step 3 examined the relationship between unsettling events and helplessness (path a). Step 4 examined the relationship between unsettling events and helplessness while controlling for sleep problems (path a’). Path a is the total effect, path a’ is the direct effect, and path b x c is the indirect effect (i.e., an exposure to unsettling events is associated with sleep problems, leading to feeling of helplessness). We then calculated the mediation analyses with nonparametric bootstrap, with 1000 resamples to obtain the proportion mediated, the magnitude of the average total effect, and the significance of the indirect effects^20^. Descriptive and regression analyses were run in SAS version 9.4 (SAS Institute Inc., Cary, NC, USA). Mediation analyses were performed by using ‘Mediation’ package in R. The significance level was set at *p* < 0.05. We followed the STROBE criteria for presenting cross-sectional data.

## Results

The present investigation is based on a sample of 1139 (86.6%) participants who reported personal treatment experiences with Covid-19 patients. Among these, n = 553 (84.8%) worked in PPs and n = 586 (88.3%) in HPs. A total of 720 (54.7%) women and 596 (45.3) men were included with more female physicians (53.2%; n = 353) working in HPs. Professional experience in patient care was high: only about 16% of participates reported < 5 years of medical employment. The majority of 98% of all physicians were vaccinated against SARS Cov2. Approximately 9% of the physicians in PPs and 15% of HP physicians had already been infected with Cov2.

### Work organisation and ethical values

Table 1 displays the impact of Covid-19 cases on the professional life of physicians, stratified for physicians working either in PPs or HPs. A high proportion of physicians in PPs (60.8%) had been confronted with dying patients. Still, confrontation with death was substantially more intense among physicians in HPs.

**Table 1:** Impact of COVID-19 cases on treatment and professional life of physicians, stratified for physicians in private practice (PPs) (n = 652) and hospital (HPs) settings (n = 664) in N (%) (Total, N = 1316).

	Total	PPs (49.5%, n = 652)	HPs (50.5%, n = 664)	*p*	*p* (male)	*p* (female)
Experience with COVID-19 patient treatment? (yes)	1139 (86.6)	553 (84.8)	586 (88.3)	0.07	0.89	**0.02**
How many of your COVID-19 patients died?						
No one	414 (36.3)	217 (39.2)	197 (33.6)	** < .0001**	** < .0001**	** < .0001**
1–9	520 (45.6)	278 (50.2)	242 (41.2)			
10–19	117 (10.3)	43 (7.8)	74 (12.6)			
≥ 20	90 (7.9)	16 (2.9)	74 (12.6)			
Cardiac co-morbidity of COVID-19 patients (yes)	617 (54.1)	271 (48.9)	346 (59.1)	**0.001**	**0.05**	**0.004**
Did the pandemic result in professional restrictions? (yes)	1059 (80.5)	521(79.9)	538 (81.0)	0.61	0.65	0.3
Did Covid-19 limit treatment of non-Covid-19 patients?						
No restrictions	168 (12.8)	87 (13.3)	81 (12.2)	0.09	0.32	0.19
Somewhat restricted	733 (55.7)	378 (58.0)	355 (53.5)			
Severely restricted	415 (31.5)	187 (28.7))	228 (34.3)			
Did you face economic disadvantages by the COVID-19 Pandemic?						
No	780 (59.4)	331 (51.0)	449 (67.6)	** < .0001**	**0.0002**	** < .0001**
Yes, moderate	409 (31.2)	257 (39.6)	152 (22.9)			
Yes, severe	124 (9.4)	61 (9.4)	63 (9.5)			
Were yourself affected by COVID-19? (yes)	156 (11.9)	59 (9.1)	97 (14.6)	**0.002**	**0.06**	**0.01**
Staff members resigned because of COVID-19? (yes)	206 (15.7)	48 (7.4)	158 (23.8)	** < .0001**	** < .0001**	** < .0001**
Do you consider regulatory restrictions for your institution/practice as meaningful?						
No	77 (5.9)	303 (46.7)	353 (53.2)	**0.01**	0.72	**0.001**
Yes, in part	583 (44.3)	301 (46.2)	282 (42.5)			
Yes, completely	656 (49.9)	48 (7.4)	29 (4.4)			
External constraints on your medical care which are in conflict with the code of medical ethics?						
No constraints	826 (62.8)	395 (60.7)	435 (64.6)	**0.002**	**0.01**	**0.02**
One time	107 (8.1)	44 (6.8)	63 (9.5)			
Several times	306 (23.3)	161 (24.7)	145 (21.8)			
On a regular basis	76 (5.8)	51 (7.8)	25 (3.8)			
Could you maintain the dignity of your patients during the pandemic? (yes)	821 (62.4)	476 (73.0)	345 (52.0)	** < .0001**	** < .0001**	** < .0001**
Are you vaccinated against SARS Cov2? (yes)	1286 (97.7)	637 (97.7)	649 (97.7)	0.96	0.26	0.22
Will COVID19 Pandemic have a lasting effect on medical care? (yes)	1065 (80.9)	535 (82.1)	530 (79.8)	0.3	0.64	0.36
Will telemedicine receive an increasing meaning in medical treatment? (yes)	982 (74.6)	442(67.8)	540 (81.3)	** < .0001**	** < .0001**	** < .0001**
Will you offer telemedicine-applications yourself? (yes)	573 (43.5)	286 (43.9)	287 (43.2)	0.81	**0.003**	**0.002**
Priorities in treatment decisions (yes)	342 (26.0)	76 (11.7)	266 (40.1)	** < .0001**	** < .0001**	** < .0001**

*p value for differences between PPs and HPs; *p* values obtained from chi-square-test for categorical variables and Wilcoxon–Mann–Whitney test for continuous variables.

Significant values are in [bold].

Job content and work organisation: Approximately 80% of all physicians experienced professional restrictions through the pandemic and about 87% reported limitations in the treatment of non-Covid-19 patients irrespective of working in PPs or HPs. Regulatory hygiene measures to ensure a personal protective environment were implemented in the majority of workplaces. More physicians in PPs than in HPs faced economic disadvantages by the pandemic (49 vs. 42.4%, *p* < 0.0001) while a fourfold higher number of staff members in HPs compared to PPs had resigned because of the pandemic (23.8 vs. 7.4%, *p* < 0.0001).

Ethical values: Covid-19 created profound conflicts between professional and ethical values for a significant proportion of participants: more physicians in PPs than HPs reported external constraints on their medical care being in conflict with the code of medical ethics (39.1 vs. 34.4%, p < 0.002). However, significantly more HP physicians were unable to maintain the dignity of their patients during the pandemic (48 vs. 27%, *p* < 0.0001).

Adverse structural impact of the medical job content: The comprehensive sum score of all adverse structural impact of Covid-19 on the work environment revealed an overall higher mean score level (mean, ± SD) for physicians in HPs compared to PPs, with no sex differences: 7.1 (3.0) in PPs and 8.4 (3.7) in HPs (*p* < 0.0001).

Future outlook: The majority of physicians (80%) believed that the Covid-19 Pandemic will have a lasting effect on medical care and about 43% will actively offer telemedicine-applications.

### Mental health of physicians during the pandemic

#### Prevalence of mental health disruptions among physicians

The majority of about 84% of physicians in PPs and HPs reported that encounters with Covid-19 patients had afflicted them disclosing a severe stressful impact of Covid-19 on the physicians’ mental state. As further displayed in Table 2, the prevalence of sleeping disturbances with 52% was high and disclosed no differences between PPs and HPs. Mean values of future expectancy ranging from a possible catastrophic to an excellent outlook of the physician’s personal future (VAS scale 1–10) revealed a minor right sided (favourable) distribution with a mean of 6.79 (± 1.88) for PPs and 6.84 (± 1.68) for HPs (n.s.). A total of 179 (13.6%) physicians suffered from clinically significant levels of depressive symptoms (Table 2)—substantially more among physicians in HPs (27.2%) than in PPs (18.7%). Findings for anxiety were similar: 317 (24.2%) in the total sample and 21.9% in PPs and 26.3% in HPs. Differences for female physicians in both settings did not reach significance.

**Table 2:** Impact of COVID-19 cases on mental distress in physicians, stratified for physicians in private practice (PPs) (n = 652) and hospital (HPs) settings (n = 664) in N (%).

	Total	PPs	HPs	*p*	*P* (male)	*P* (female)
		(49.5%, n = 652)	(50.5%, n = 664)			
Have encounters with COVID-19 Patients unsettled yourself?						
This was not the case	210 (16.0)	107 (16.4)	103 (15.5)	**0.005**	**0.02**	0.14
Somewhat	352 (26.8)	203 (31.1)	149 (22.4)			
Moderate	307 (23.3)	142 (21.8)	165 (24.9)			
Fairly	299 (22.7)	133 (20.4)	166 (25.0)			
Very much	148 (11.3)	67 (10.3)	81 (12.2)			
Have encounters with COVID-19 Patients unsettled yourself?						
A little/No	562 (57.3)	310 (47.6)	252 (38.0)	**0.0004**	**0.01**	**0.01**
Moderate/Fairly/Very	754 (42.7)	342 (52.5)	412 (62.1)			
Feeling helpless	768 (58.4)	348 (53.4)	420 (63.3)	**0.0002**	**0.0001**	0.1
Self-rated health						
Poor	31 (2.4)	17 (2.6)	14 (2.1)	0.5	0.45	0.97
Moderate	201 (15.3)	106 (16.3)	95 (14.3)			
Good	570 (43.3)	289 (44.3)	281 (42.3)			
Very good	398 (30.2)	188 (28.8)	210 (31.6)			
Excellent	116 (8.8)	52 (7.9)	64 (9.6)			
Self-rated health						
Poor	232 (17.6)	123 (18.9)	109 (16.4)	0.24	0.21	0.65
Good	1084 (82.4)	529 (81.1)	555 (83.6)			
How would you rate your future? 1 catastrophal 10 excellent (Mean, ± SD)	6.82 (± 1.79)	6.79 (± 1.88)	6.84 (± 1.68)	0.004	0.7	0.61
Sleep disturbances						
No	635 (48.3)	312 (47.9)	323 (48.6)	0.005	**0.01**	**0.04**
Difficulties initiating sleep	130 (9.9)	48 (7.4)	82 (12.4)			
Difficulties maintaining sleep	362 (27.5)	184 (28.2)	178 (26.8)			
Waking up too early	189 (14.4)	108 (16.6)	81 (12.2)			
Sleep disturbances						
No	635 (48.2)	340 (52.2)	341 (51.4)	0.77	0.08	0.06
Yes	681 (51.8)	312 (47.9)	323 (48.6)			
Depression (Yes)	302 (23.0)	122 (18.7)	180 (27.2)	**0.0002**	**0.0006**	0.08
Anxiety (Yes)	317 (24.16)	143 (21.9)	174 (26.3)	0.07	**0.004**	0.99
How difficult have problems made it for you to do your work, take care of things at home, or get along with other people?						
Not difficult at all	581 (44.2)	290 (44.5)	291 (44.0)	0.07	0.18	**0.05**
Somewhat difficult	543 (41.3)	284 (43.6)	259 (39.1)			
Very difficult	139 (10.6)	57 (8.7)	82 (12.4)			
Extremely difficult	51 (3.9)	21 (3.2)	30 (4.5)			

*p value for differences between PPs and HPs; *p* values obtained from chi-square-test for categorical variables and Wilcoxon–Mann–Whitney test for continuous variables.

Significant values are in [bold].

Applying mean PHQ-2 and GAD-2 data from reference groups and population based studies before and in the early stages of the Covid-19 pandemic, Table 3 reveals significant higher values compared to earlier studies among physicians (A and B) but lower values compared to a population based study in the early phase of the pandemic (C). However, mean values compared to a pre-pandemic population based screening study (D) were substantially higher.

**Table 3:** Severity of depressive symptoms (PHQ-2) and generalized anxiety symptoms (GAD-2) for physicians in private practice and hospital settings, in comparison with general population and reference groups before and during the COVID-19 pandemic.

Study population	Mean (± SD)	N	Comparison with physicians during the COVID-19 pandemic in Germany (N = 1061)^a^		Comparison with physicians during the early phase of pandemic in Germany (N = 492)^b^			Comparison with general population during early phase of pandemic in Germany (N = 6509)^c^		Comparison with general population in Germany (N = 5010)^d^		
			Mean (± SD)	*P*; effect size	Mean (± SD)	*P*; effect size		Mean (± SD)	*P*; effect size		Mean (± SD)	*P*; effect size
Depressive symptoms (PHQ-2)												
Total	1.76 (± 1.50)	1313	1.48 (± 1.35)	< .0001; 0.20	0.60 (± 1.13)		< .0001; 0.82	2.11 (± 1.70)	< .0001; 0.56		0.94 (± 1.20)	< .0001; 0.65
Private physicians	1.61 (± 1.42)	652		0.02; 0.10			< .0001; 0.78		< .0001; 0.30			< 0.0001; 0.55
Hospital	1.91 (± 1.56)	661		< 0.0001; 0.30			< .0001; 0.94		0.01; 0.12			< 0.0001; 0.78
Generalized anxiety symptoms (GAD-2)												
Total	1.66 (± 1.52)	1314	1.45 (± 1.41)	< .0001; 0.14	–		–	2.03 (± 1.76)	< .0001; 0.22		0.82 (± 1.10)	< .0001; 0.70
Private physicians	1.60 (± 1.45)	652		0.01; 0.01					< 0.0001; 0.25			< 0.0001; 0.68
Hospital	1.72 (± 1.58)	662		< 0.0001; 0.18					< 0.0001; 0.18			< 0.0001; 0.77

^a^Morawa et al.^21^.

^b^Skoda et al.^22^.

^c^Petzold et al.^23^.

^d^Löwe et al.^24^.

*Mean (± SD) and pairwise comparison, *P* values; effect size Cohen’s d; d ≥ 0.2 = small, d ≥ 0.5 = medium and d ≥ 0.8 = large effect size.

#### Covariates of helplessness

**Figure 2:**
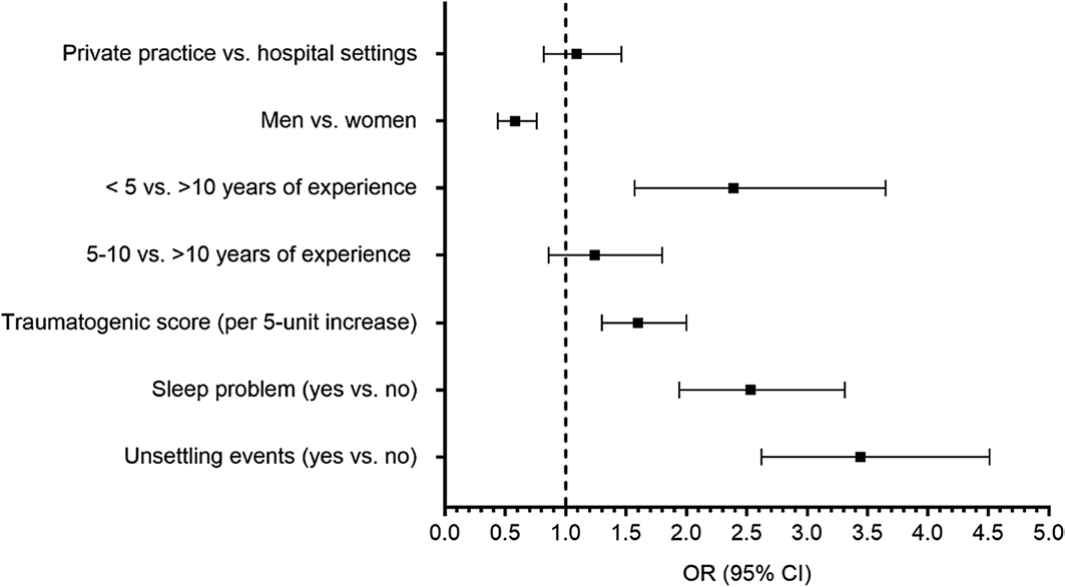
Factors associated with helplessness (feeling helpless vs. none) in physicians during the fourth wave of COVID-19 pandemic (N = 1315). *Depicted are Odds Ratios (OR), 95% Confidence Intervals (CI) and *P* values estimated from multivariate logistic regression.

A total of n = 768 (58.4%) of participants reported feelings of helplessness with significantly more physicians in HPs (420, 63.3%) than in PPs (348, 53.4%). Covariates significantly associated with feeling helpless are displayed in Fig. 2: male sex (OR = 0.58, 95% CI = 0.44–0.76) was associated with lower odds of helplessness, while having < 5 years of experience (2.39, 1.57–3.65), sleeping problems (2.50, 1.94–3.30), traumatogenic score (per 5-unit increase) (1.60, 1.30–2.00) and being encountered to an unsettling event (3.44, 2.62–4.51) were associated with higher odds of helplessness.

#### Mediation analysis

**Figure 3:**
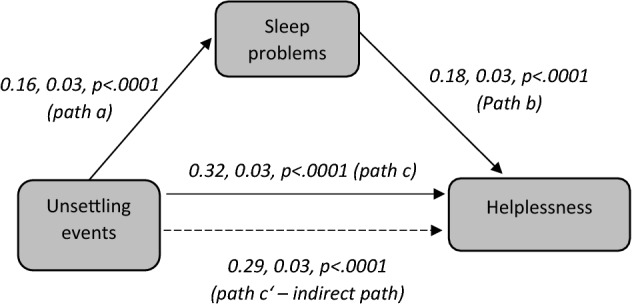
Mediation analysis of sleep problems on the association between unsettling events and helplessness. Graphical representation of the mediation analysis: *Path a* probes the relationship between unsettling events and sleep problems. *Path b* probes the relationship between sleep problems and helplessness, while controlling for unsettling events. *Path c* probes the relationship between unsettling events and helplessness. *Path c’* probes the relationship unsettling events and helplessness, while controlling for sleep problems. ß estimates with standard errors and *p* values from sex-adjusted regression models are reported for each association examined.

In a sensitivity analysis as depicted in Fig. 3, the association between exposure to unsettling events and helplessness was significantly mediated by sleep disturbances (Indirect path: ß = 0.29, SE = 0.03, *p* < 0.0001).

## Discussion

The Covid-19 pandemic has burdened an unprecedented psychological stress on members of the medical workforce—in the initial phase especially on frontline medical staff in close contact with infected patients. With the passage of time, professional involvement with Covid-19 patients generalized to a much wider array of medical disciplines without being sufficiently addressed in research attempts. Focussing on physicians with treatment experiences of Covid-19 patients either working in PPs or in HPs, we were able to show that the pandemic emerged as a sustained crisis situation with a major structural impact and psychological challenges for physicians in both settings which remained being present during the culmination point of the fourth Covid-19 pandemic in late 2021.

### Workplace aspects

Of note, physicians were exposed to a multitude of challenging or even frightening aspects of their work environment regardless of working in PPs or HPs. Often confronted with dying Covid-19 patients; with professional restrictions including limitations in the treatment of non-Covid-19 patients; with facing pandemic induced economic disadvantages and being subjected to limiting hygiene measures in their practice/institutions. Most importantly, however, Covid-19 created profound conflicts between professional and ethical values for a significant proportion of all participating physicians. Although more physicians in PPs than HPs reported external constraints on their medical care being in conflict with the code of medical ethics, substantially significantly more HP physicians were unable to maintain the dignity of their patients during the pandemic.

Taken together, these adverse structural features of Covid-19 on the work environment constitute a universal hazard for the medical work force irrespective of the particular setting^25^. The high impact of disturbing features on physicians has not been shown before and not been addressed particularly comparing physicians working in PP and HP settings. It becomes evident that the level of distressing effects has reached a substantial impact on the professional life of all physicians involved in Covid-19 treatment (although still more intense for physicians working in HPs).

### Mental health of physicians treating Covid-19 patients

*Depression and anxiety:* Umbrella review data derived from early stages of the pandemic^4^ point to an excess prevalence of approximately 40% for depression and 17% for anxiety among physicians. A more recent meta-analysis among doctors during the first twelve months of the Covid-19 pandemic, calculated from 26 studies and 31,447 participants, found a significantly lower and more realistic pooled prevalence of depression of 20.5% (95% CI 16.0–25.3%). The same is true for anxiety: here, they found a higher overall pooled prevalence in 25.8% (95% CI 20.4–31.5%) of physicians, calculated from 30 studies including 33,281 participants^26^. In our study with data assessed during the culminating point of the fourth wave of the pandemic, 23.0% of all physicians under investigation suffered from clinical meaningful depressive symptoms and thus were in the upper range of prevalence estimates from the later meta-analysis. However, stratification between physicians in PPs and HPs disclosed a marked difference: 18.7% in PPs but a much higher total of 27.2% in HPs experienced depression. A comparable picture appeared for anxiety: 24.16% of all participants stratified for 21.9% in PPs and 26.3% in HPs reported significant anxiety.

In the early phases of the pandemic, a steep incline in depression and anxiety had been observed compared to the pre-pandemic time. Repeated cross sectional findings from representative surveys in the US (e.g.^27,28^ and elsewhere^23^, or longitudinal data from the UK^29^ point to an approximately threefold increase in the number of people with meaningful severity of depressed symptoms during the initial phase of the pandemic. Comparing mean values of depression and anxiety with pre- Covid-19 data from the general population in Germany (N = 6509) (with the identical screening instrument) revealed that the means had doubled^24^.

The question arises whether over the passage of time, a downward trend from these early peaks may have occurred particularly among health specialists. After experiencing an initial shockwave, so the idea, people may have adapted to the stressful global situation^30^. The German web-based VOICE survey including data from 1.006 HPs performed during the early phase of the pandemic allows an adequate comparison^21^: at that time point, a significantly lower prevalence of 17.4% for clinically significant levels of depressive and 17.8% for anxiety symptoms (assessed with the identical screening instrument as in our study) were documented (see also^22^). Taken together, our data do not support the assumption that over the passage of time, a downward trend from the early peaks has occurred. On the contrary: our data indicate a further increase in affective suffering among physicians, particularly in HPs.

*Helplessness:* Helplessness is a cognitive state often combined with desperate affective sentiments in the face of losing control over a given situation or its outcomes. The exponent is increasingly convinced that there is nothing that can be done to improve the hopeless situation. It is of particular concern that more than half of all physicians (58.4%) confirmed feeling helplessness—a psychological condition which may (at least gradually) hamper the exponent’s ability to function properly and may in part inhibit engagement in new, potentially effective, behaviours—qualifications, which are intuitively expected being a core feature of the medical profession. Thus, the high level of helplessness among physicians is likely to reflect the enormous threat the pandemic unfolds on medical professionals. Being encountered to an unsettling event (3.4-fold) had the strongest impact in odds of feeling helpless. Physicians being junior in terms of their position at work (evidenced as being in the lowest stratum of medical experience), female sex and sustained sleeping disturbances were less likely to resist to the toxic impact of the sustained traumatising work environment^7^. Sleep disturbances in particular not only reflect a cumulative number of worries and experience of adversities 22 but may also weaken the capacity to withstand stressful hardship. Based on these findings, it is not surprising that significantly more physicians in HPs (63.3%) than in PPs (53.4%) experienced these feelings.

Our study disclosed features which contribute to helplessness among physicians. It confirms that being encountered to an unsettling event (3.4-fold increase in odds of feeling helpless) had the strongest impact. Physicians in the lowest stratum of medical experience, female sex and sustained sleep problems were less likely to resist to the toxic impact of the sustained traumatising work environment. Sleep problems in particular not only reflect a cumulative number of worries and experience of adversities^31^ but may also weaken the capacity to withstand stressful hardship. Based on these findings, it is not surprising that significantly more physicians in HPs (63.3%) than in PPs (53.4%) experienced these feelings.

Female gender emerged as a solid risk factor for psychological deterioration among physicians being exposed to Covid-19 patients. This finding is in line with evidence from a multitude of earlier studies and meta-analyses^3,7,32^. Interestingly, Lasalvia et al.^11^ calculated differential risks of distress sub-conditions and disclosed an adjusted OR for PTSD in female physicians compared to male counterparts of 1.34, for anxiety of 2.18 and for depression of 1.70. Although the gender gap has been confirmed as a stable finding, clear explanations are missing. Following evidence from a recent clinical report^33^, it is not unlikely that female physicians employ a more compassionate and caring attitude towards the patients—a favourable mindset for which being more severely encountered and worried may be the toll female physicians have to pay.

### Interventions

The enormous level of emotional and psychological suffering due to the multitude of threatening factors induced by the Covid-19 pandemic both in the PP and in the HP setting urgently call for implementation of preventive measures. Kisely et al.^7^ compiled evidence based measures which can be employed in HP settings. As organisational factors that are likely to decrease risk of adverse psychological outcomes, they recommend among others frequent short breaks from clinical duties; adequate time off work; supportive peers, positive feedback to staff, effective staff training in preparation for outbreaks; staff support protocols, and clear communication with staff. For physicians in PPs the organisational backup is much less advanced and support from the peer group is sparce. Many PP physicians are forced on self-perception of being adequately trained, to have faith in precautionary measures and also to profit from family support.

For both settings, the access to tailored psychological interventions based on needs of the particular burdensome conditions caused by Covid-19 is an option. For such approaches, the usefulness of a compassion training for physicians has been advocated by revealing the potential use of these approaches in the preventive occupational assessment for professional hazards^34^.

### Limitations

One strength of the present investigation is the focus on physicians in HPs and in PPs with an in-depth assessment of disturbing medical job contents which were related to mental health impairments of the physicians involved. The major limitation is the low response rate increasing a sampling bias due to respondents’ self-selection to participate. However, following a decision framework for assessing nonresponse bias^35^, we found other investigations with involvement of physicians from the early phase of the pandemic undertaken with identical methods and comparable features (e.g. participation rates, sex distribution, amount of medical experience of participating physicians). These study features also helped to overcome the limitations of a cross sectional design by allowing estimates of repeated cross sectional findings. However, the cross-sectional study prevents knowledge about causal relationships. Furthermore, self-report bias may be present as the major content of the study was assessed with self-reporting questionnaires^36^.

## Conclusions

During the culminating point of the fourth wave of the pandemic, 23% of physicians under investigation suffered from clinical meaningful depressive symptoms and 24% from anxiety. Comparisons with cross sectional studies of physicians during the early phase of the pandemic suggest an increase of affective burden and do not support a healing adaptation to the adverse environment. The results provide new insights by evidencing that for physicians working either in HP or in PP settings, Covid-19 plays a crucial role in exacerbating mental health impairments and declines in quality of life. For a substantial proportion of physicians under investigation the pandemic cause feelings of helplessness which may compromise in part the ability of the exponents to function properly in the sustained traumatizing content.

## Data Availability

Data sharing for collaborative work is highly welcomed. Individual participant data and a data dictionary defining each field in the set will be made available to others after approval of a proposal with a signed data access agreement sent to the Academic research organisation Atrial Fibrillation NETwork (AFNET), Münster, Germany. info@af-net.eu.
